# The Frequency of Clinic Visits Was Not Associated with Medication Adherence or Outcome in Children with Inflammatory Bowel Diseases

**DOI:** 10.1155/2018/4687041

**Published:** 2018-02-25

**Authors:** Cheryl Kluthe, Jenkin Tsui, Donald Spady, Matthew Carroll, Eytan Wine, Hien Quoc Huynh

**Affiliations:** ^1^Edmonton Pediatric IBD Clinic (EPIC), Edmonton, AB, Canada; ^2^University of Edinburgh, Edinburgh, UK; ^3^Division of Pediatric GI Nutrition, Stollery Children's Hospital, University of Alberta, Edmonton, AB, Canada

## Abstract

**Background:**

Medication nonadherence is a challenge in pediatric patients with inflammatory bowel diseases (IBD). Poor adherence can result in disease flare-ups, disease complicationstherapy escalation, and the need for corticosteroids. The aim was to determine if clinic visit frequency was associated with treatment adherence.

**Methods:**

A retrospective chart review of patients attending the Edmonton Pediatric IBD Clinic (EPIC) at the Stollery Children's Hospital from January 2012 to December 2013 was completed. Correlations were made between frequency of clinic visit, percentage of prescriptions filled, percentage of requisitioned blood work completed, rural or urban residence, and steroid-free remission status of patients for the 6 months after the chart review.

**Results:**

127 patients were reviewed with 82 patients diagnosed with Crohn's disease (CD) and 46 with ulcerative colitis (UC) which included one IBD-Unclassified. Mean age at diagnosis is 9.17 years and median duration of follow-up is 3.2 years. Almost all patients on infliximab infusions received them “within window.” Immunomodulator median adherence rate was 88%. 5-ASA adherence was 82%. A median of 67% of patients had blood work completed as requested. Clinic visit frequency was not associated with adherence to blood work or to medications. Duration of disease was the only independent factor found to be associated with a reduction in blood work and immunomodulator adherence (“OR 0.86 and 95% CI: 0.74–0.99” and “OR 0.82 and 95% CI: 0.71–0.97”) per year, respectively. Patients who remained corticosteroid-free in the 6 months after the 2 years' adherence review had an overall median medication adherence rate of 86% compared to only 53% for those who relapsed and required corticosteroids (*p* = 0.01).

**Conclusion:**

Clinic visit frequency was not associated with patient adherence to medications or blood work. However, disease duration was found to be associated with medication adherence. Adherent patients were more likely to remain in steroid-free remission.

## 1. Introduction

### 1.1. Background

Inflammatory bowel disease (IBD) is a chronic condition that leads to inflammation in the digestive tract. It is characterized by periods of disease activity and periods of disease remission [1]. Canada has one of the highest incidence rates of Crohn's disease (CD) and ulcerative colitis (UC) in the world: 13.4 and 11.8 cases over 100,000 persons for CD and UC [2]. Furthermore, the incidence of IBD in children under 10 years of age is increasing [3]. Currently, there is no cure for IBD, leading to the need for chronic treatment. Medication nonadherence is a challenging issue for patients with a chronic disease, including pediatric patients with IBD. Pediatric IBD is unique in that both the patients and the parents have a shared responsibility for administering medications [4]. Poor adherence can result in disease flare-ups, disease complications, therapy escalation, and need for corticosteroids. Previous studies indicate that nonadherent patients are 5.5 times more likely to experience a flare compared to individuals who are adherent [4]. Nonadherence rates range from 50 to 80% [4], with patients overestimating their oral medication intake rate by 23% [5]. Reasons often given for poor adherence include the following: forgetfulness, patient perceptions of side effects, and lack of education [6].

It has been shown that, by improving a patient's adherence rate, they can potentially decrease disease activity and improve long-term outcomes. Physicians commonly provide knowledge and education to their patients about disease management; however ultimately it is up to the patient to follow this advice. One factor in a patient's care that the physician can influence is the frequency of scheduled follow-up visits. The goal of this study was to investigate the association between frequency of clinic follow-up on adherence to medications and blood work and treatment outcomes.

## 2. Methods

### 2.1. Study Design

A retrospective chart review of patients attending the Edmonton Pediatric IBD Clinic (EPIC) at the Stollery Children's Hospital between January 2012 and December 2013 was conducted utilizing the EPIC research registry, “Global Outcomes in IBD.” Written informed consent was obtained from all parents/legal guardians, and assent was obtained from children. Ethics approval was obtained from the University of Alberta Research Ethics Board.

*Inclusions Criteria*. Patients age must be between 2 and 17 years at time of diagnosis. IBD was diagnosed using standard criteria based on symptoms, endoscopy, and imaging findings and have at least a year of follow-up.

*Exclusion Criteria*. Patients who have less than 1 year of follow-up, patients listed for liver transplant (as they were also being followed by hepatologists), and patients living outside of the province of Alberta (their prescription and blood work data were not available on the provincial database) were excluded.

### 2.2. Variables

Information regarding disease diagnosis, age, gender, place of residence, baseline disease activity [Pediatric Crohn's Disease Activity Index (PCDAI) and Pediatric Ulcerative Colitis Activity Index (PUCAI)], and pharmaceutical managements were recorded for all eligible patients. Place of residence was broadly categorized as urban or rural using postal code (as specified by Census Canada), except for St Albert, Spruce Grove, Fort Saskatchewan, and Sherwood Park. Patients from these communities are adjacent to the immediate city limits of Edmonton and were therefore considered urban given their increased access to healthcare services. For the interim years (between time of diagnosis and 2012), any significant event such as flares, hospitalizations, or surgeries were recorded, as well as any change in maintenance therapy.

### 2.3. Data Source/Measurement

For the years 2012 and 2013, the frequency of clinic visits was extracted along with the corresponding clinical disease activity scores (PCDAI or PUCAI) for each visit. Disease flares were defined as any disease activity above a patient's normal status (i.e., PCDAI of >10 or PUCAI score > 10 compared to previous visit). In addition, any significant event such as emergency room visits, hospitalizations, or surgeries due to IBD were recorded. All IBD related medications (induction therapy and maintenance therapy) were noted along with any changes made to prescriptions during the study period. Using the Alberta provincial pharmacy database, all dispensed medications were recorded (initial dispensing plus refills). Episodes of laboratory investigations (individual blood work encounters) were determined using the Alberta Netcare electronic health record which collates all results from investigations performed in the province. The number of requisitioned laboratory encounters was determined by reviewing the electronic orders of individual patient encounters. This allowed “routine” laboratory encounters to be captured in addition to “unscheduled” encounters, such as those that may have been requested during a disease flare-up. For “routine” lab work, patients are given “standing order” blood work requisitions in clinic which specify frequency, thus obviating the need for new requisitions each time blood work is required.

### 2.4. Medication Adherence Rate

A medication adherence rate as a proportion was determined for each patient by comparing the number of prescriptions filled (days of medication dispensed) against the number of days of the year that a patient should have been taking the prescribed therapy over a 1-year period for both 2012 and 2013. This was a continuous variable determined for each patient.

#### 2.4.1. Medication Adherence

As in previous studies, the medication adherence was defined as a patient having filled 80% or more of the prescribed medication [4]. This allowed adherence to be treated as a dichotomous variable in statistical analyses. Adherence to the antitumor necrosis factor*α* (anti-TNF*α*) therapy infliximab was defined as having received the medication “within window” (i.e., the prescribed frequency plus or minus 3 days). Nonadherence to infliximab was defined by a discrepancy of >72 hours between the scheduled date of infusion and the actual date of administration [7]. As stated by Ma et al., there is no widely accepted definition for adherence to an individual infliximab infusion. A 72-hour cut-off period was empirically decided on (between 3 days before and 3 days after their scheduled infusion date) as it was felt that this reflected a sufficient time to accommodate weekends, statutory holidays, infusion clinic availability, and minor personal reasons that may briefly delay or expedite the infusion schedule [7]. Any infusion given more than 3 days late was considered “out of window.” If infliximab was administered early, held, or postponed under physician advice, it was not considered to be “out of window.” No definition for Adalimumab “within window” was needed due to the extremely small number of patients receiving this subcutaneous form of anti-TNF*α* therapy.

### 2.5. Blood Work Adherence Rate

This was determined by comparing the number of completed laboratory encounters against the requisitioned number of encounters over a 1-year period for both 2012 and 2013. Patients are given “standing order” blood work requisitions in clinic with specify frequency, thus obviating the need for a new requisition each time blood work is required.

#### 2.5.1. Steroid-Free Remission

Steroid-free remission was defined as the absence of enteral or parenteral corticosteroid use during the specified time period for patients who remained in clinical remission. After completion of primary data collection (December 2013), a further prospective 6-month follow-up period was performed to capture any patients who may have required courses of corticosteroids beyond the defined study period.

Data were analyzed looking at the population as a total group (all IBD) and then stratified by disease type (CD and UC). IBD-U was combined with UC. The following variables were assessed to determine association with adherence: clinic frequency, age, sex, address, disease type, severity of disease activity at diagnosis, duration, and exacerbations during the period of assessment (flares, hospitalization, or surgeries).

To analyze the effect of frequency of clinic visits on outcomes, we used both number of clinic appointments per year as a continuous variable and grouping patients into 3 categories based on the number of times they were seen in clinic per year: 0-1 time, 2-3 times, and 4 times or higher. These three categories were arbitrarily chosen but they correspond to standard practice of EPIC, where minimal follow-up would be considered 0-1 times, standard follow-up would be 2-3 times, and frequent follow-up would be 4 or more times per year.

## 3. Statistical Methods

### 3.1. Study Size

Study size was calculated using the University of British Columbia (UBC) power calculator inference for proportions (http://www.stat.ubc.ca/~rollin/stats/ssize/b2.html). An estimated adherence rate of 80% for frequently seen patients was utilized based on previous publications (Kitney et al.) whilst those seen infrequently were estimated to have an adherence rate of 55%. Using a 2-sided test, alpha cut-off of 0.05, we calculated a required sample size of 110 patients to achieve 80% power.

Categorical variables were expressed as proportions and compared using Chi square analysis. Continuous variables were calculated and reported as mean and standard deviation or median and interquartile range as indicated by their distribution. Correlations between primary variables were calculated using Pearson's correlation whilst nonparametric comparisons of continuous variables were calculated using the Mann–Whitney *U* test. To deal with the repeated outcome measures over the two-year study period and the problem of within-subject correlations, a generalized estimating equation (GEE) model was used. Repeated observations on the same data unit collected over successive time points were correlated over time. The GEE method accounts for this correlation using a semiparametric approach to such longitudinal analysis of categorical data. As the outcome variables were proportions type of data including 1 (or 100%), linear regression (1-inflated Beta regression when necessary) was performed. Analyses were conducted using Stata 13.1 software (StataCorp, LP, College Station, Texas). STATA/IC version 13.2 was used for the regression analysis. A *p* value less than 0.05 was considered statistically significant.

## 4. Results

### 4.1. Participants

One hundred and twenty-seven patients were reviewed; 81 patients were diagnosed with Crohn's disease (CD) and 46 with ulcerative colitis (UC) which included 1 IBD-Unclassified (Table 1). Patient demographics and disease characteristics and medication treatment were further described in Table 1. Sixty-five percent had an urban residence. Both diseases had very similar follow-up of over 3 years. Nearly 64% of patients were seen in clinic 4 or more times, 21.6% were seen 2 to 3 times, and 14.6% were seen only 0 to 1 time per year. Forty-three percent of UC patients were treated with 5-ASA alone versus 2% for CD. Immunomodulators were used in 67% of all patients. Methotrexate (MTX) was used in 55% of males and Azathioprine (AZA) was used in 83% of females (*p* = 0.002). Thirty-seven percent of patients were on immunomodulators only. Dual therapy with immunomodulators and anti-TNF*α* preparations (IFX or Adalimumab) were used in 25% of patients in 2012; this increased to 44% in 2013. Monotherapy, either with IFX or ADA, was used in 8 (6%) patients.

### 4.2. Medication and Blood Work Adherence Rates and Clinic Visits

Table 2 reviews the adherence rates to 3 key maintenance medications for IBD patients and their adherence to blood work. Only 3 patients were receiving Adalimumab. Almost all patients treated with anti-TNF*α* received infliximab and were infused within window (median of 100%). Patients taking immunomodulators had a median adherence rate of 88%; 5-ASA had the lowest median adherence rate of 82%. A median 67% of patients completed the blood work that was ordered.

**Figure 1:**
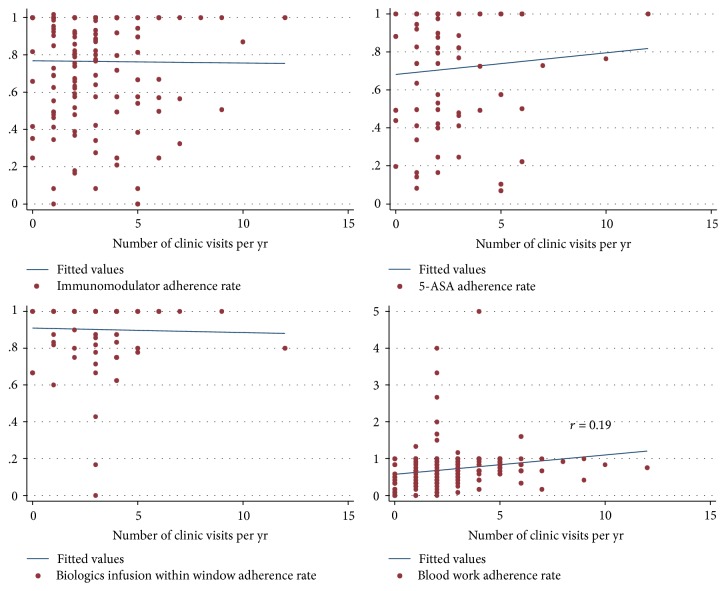
Lack of correlation between adherence rate and clinic visits.

Before categorizing the adherence rate and clinic visits, analysis in Figure 1 showed that there was no correlation between the adherence rates of immunomodulators, 5-ASA, and IFX infusion within window and number of clinic visits per year. There was a minimal correlation between blood work adherence and clinic visit frequency (*r* = 0.19 and *p* = 0.4).

Further analysis was carried out using conventional definition of adherence defined as filling 80% or more of the prescriptions or requisitioned blood work prescribed (Tables 3(a) and 3(b)). For anti-TNF*α*, adherence was defined as >80% given within window.  Table 3(a) describes the categorical independent variables and Table 3(b) describes continuous independent variables in relation to adherence to immunomodulators, 5-ASA, and biologics as well as blood work adherence. Clinic visits were described in both tables as both continuous and categorical variables. The number of observations refers to the number of subjects that took the medication per year for 2 years (2012 and 2013).  Table 4(a) describes the univariate analysis of the factors that could affect adherence. Overall age, gender, disease type, and disease exacerbation (flare, surgery, and hospitalization) during the influence period did not influence patient medication adherence rates. Frequency of clinic visits did not appear to be associated with adherence to medications or blood work when clinic visits were analyzed as either a categorical or continuous variable.

Blood work adherence appeared to be associated with older age of diagnosis, shorter duration of disease, and exacerbation of disease during study period and more frequent clinic visits on univariate analysis. However, on multivariable analysis (Table 4(b)), only duration of disease was an independent factor that was associated with blood work adherence with a reduction of 14% per year (OR 0.86, 95% CI: 0.74–0.99), confirming the minimal correlation (*r* = 0.19) seen between blood work adherence rate and number of clinic visits per year in Figure 1. This correlation was confounded by duration from time of diagnosis.

Shorter duration from time of diagnosis also remained an independent factor associated with adherence to immunomodulators. With each year away from time of diagnosis, the odds ratio was reduced (OR 0.82, 95% CI: 0.71–0.97).

About three-quarters of patients taking 5-ASA had a diagnosis of UC. Disease severity and urban living were independent factors associated with 5-ASA adherence, for moderate disease when compared to mild disease (OR 3.2, 95% CI: 1.09–9.53) and urban patients when compared to rural patients (OR 3.6, 95% CI: 1.2–11) (see Table 4(b)).

### 4.3. Steroid-Free Remission and Adherence Rate

Table 5 shows that adherence rates affected patient outcomes. Patients adhering to all medications were more likely to remain in a steroid-free remission: those who remained corticosteroid-free had an overall median medication adherence rate of 86% compared to only 53% for those who relapsed and required corticosteroids (*p* = 0.01). Patients who adhered to their immunomodulators (median of 90%) remained without corticosteroids, versus only a median of 57% of those who used corticosteroids (*p* = 0.01). In fact, none of the UC patients that were adherent to 5-ASA and immunomodulators in the preceding 6 months required corticosteroids.

## 5. Discussion

The main purpose of this study was to explore the frequency of clinic appointments on adherence rates to medications and associated outcomes in IBD patients. Clinic visit frequency was not found to be associated with medication or blood work adherence. Univariate analysis appeared to show that increased clinic frequency was associated with blood work adherence but the association became insignificant when corrected for duration of disease and age of diagnosis. The increase in frequency of clinic visits is more related to early stage of the diagnosis, when patients are more likely to adhere to their blood work requirements.

The medication adherence rates of the patients in our population are similar to those found in previous studies [4]. Among the standard maintenance IBD medications, 5-ASA medications had a lower adherence rate with a median of 82%, which can often be associated with medications that have multiple daily dosing [8]. The fact that most patients on 5-ASA monotherapy had mild UC, and thus might be less concerned about flare-ups, may explain the low 5-ASA adherence. Adherence to 5-ASA was the only medication that was independently associated with disease severity when comparing moderate disease to mild one. Adherence was also much better among urban residents. The number of patients taking 5-ASA in our cohort is small, so these results need to be interpreted with caution. These two factors severity and rural/urban living were not observed be associated with adherence to immunomodulators, anti-TNF*α*, or blood work. Studies comparing medication adherence rates between urban and rural settings for other chronic diseases have not been consistent [9]. Patients with severe disease have been shown to be more motivated to perform proper health behaviour [10].

The median adherence rate to immunomodulators was 88%. More of our patients with Crohn's disease were treated with immunomodulators than are those with UC. Anti-TNF*α* adherence, with most receiving infliximab, was excellent with virtually all patients that received infusion within window. Blood work adherence rate was the lowest in our patients with a median of 67% in outpatients.

Duration of disease was found to be an independent predictor of adherence to immunomodulators and blood work. Increased adherence is often associated with increased belief in the need for a medication; this effect gradually diminishes over time as shown in the rheumatology literature [11]. However, we found no association between duration of disease and 5-ASA or anti-TNF*α* adherence. Our study, with fewer patients taking 5-ASA, may not have been sufficiently powered to show an association between 5-ASA adherence and duration of disease. We also did not observe any association between duration of disease and biologic use. The likely explanation for this is the very high adherence rate defined as given within window in the biologic group. It was no surprise that blood work adherence was independently associated with the recently diagnosed IBD children, that is, those with short duration from diagnosis and those who had exacerbation during the study period (*p* = 0.09). We generally request more blood work for these patients. Also, it is during this initial period when patients are often the most symptomatic and are relying on their medications to get them into remission. Once remission is achieved and patients have been “well,” for a period, our data suggest that they are more likely to be nonadherent. The period of 1 to 2 years after diagnosis is an important time for medication education to occur to improve the patient's knowledge of the natural history of disease and to understand the importance of medication compliance in preventing relapses. Age, gender, and exacerbation (defined as having surgery or corticosteroid exposure due to IBD) during the study did not impact a patient's medication adherence rate.

Adherent patients are more likely to remain in a steroid-free remission in the following 6 months. This was observed in those taking immunomodulators as well as in the combining the medication adherence group. 5-ASA and anti-TNF*α* adherence was not significant in predicting steroid remission; this is again likely a reflection of low number needed for corticosteroids and the very high rate of adherence for anti-TNF*α*. Age group and gender did not have an impact. This is different from other studies, where these demographic variables were found to be associated with adherence rates [12]. Perhaps this can be attributed to the fact that we included only pediatric patients in whom it is the parents' responsibility to remind the child to take the medication.

Limitations to this study include a mixed population of eligible patients who attended the EPIC clinic including recently diagnosed patients (1 year after diagnosis), different age, and severity and chronicity of disease. Recently diagnosed patients are often more adherent to their treatment as the diagnosis is new and they are commonly experiencing symptoms. Their adherence at this time may be an unrealistic representation of what their adherence will be a few years into their disease. No socioeconomic and education status of the family was collected. The prescription refill information is available on the provincial pharmacy database; however, it is the pharmacy's responsibility to update the system with each prescription refill. In addition, there was no way to check the accuracy of the information or if information was missing.

Frequency of clinic appointments did not affect adherence rates for medications or blood work. Health care providers view clinic appointments as an excellent opportunity to reinforce education to patients and their families. Physicians may see their nonadherent patients more frequently in the hope of improving their adherence rates, but based on these data this strategy may not have the desired impact. Lack of disease education has been shown to be a barrier to medication adherence [6]; however our current way of running our IBD clinic and educating our patients through clinic appointments did not appear to improve treatment adherence. Patients on average will only remember 60% of the information that is told to them [13]. Therefore, families may only take away from the clinic appointment a few key points and forget the additional information that the doctor or nurse provided. The way we explain side effects and patient perception of side effects can also affect a patient's decision to take their medications [14]. Patients with a good relationship with the treatment team and the team's willingness to listen to the patient's concerns and involve them in the decision-making may make patients more willing to follow the treatment plan [5]. Patient preference is integral to decision-making regarding treatment decisions and can increase the success in treatment adherence and achieve a better health outcome [15].

IBD clinics should intend to develope strategies to enhance the above qualities that promote adherence. An example would be intentionally monitoring and reviewing blood work and other disease monitoring investigations with caregivers and patients at regular intervals, providing the reassurance that the medication is well tolerated and effective. The process where the patient's ability or desire to adhere to their medications when they return to their routine schedule is influenced by their environment such as family finance, beliefs about modern medicine, and how a patient fits their medications into their day-to-day life. A clear patient-specific understanding of these issues may be a better starting point to understand how healthcare providers can improve a patient's medication adherence rates.

In conclusion, medication adherence is an ongoing challenge in chronic illness, which was not associated with clinic visit frequency. It may be more likely influenced by the quality of those clinic visits. Qualitative research is required to explore the factors that really motivate patients to be adherent to their treatment plan.

## Figures and Tables

**Table 1:** Patient demographics and medication treatment.

	Total group (%)	Crohn's (%)	Ulcerative colitis (%)
Total patients	127	82 (65)	46^*∗*^ (35)
Male patient	72 (57)	49 (60)	23 (50)
Age at diagnosis in years, mean (std. Dev.)	10.38 (4.00)	11.06 (3.47)	9.17 (4.54)
Disease activity at diagnosis PCDAI for CD, PUCAI for UC Mean (std. Dev.)	NA	21.7 (11.16)	31.9 (16.56)
Duration of disease since diagnosis in years: median (IQR)	3.2 (2 to 5.9)	3.1 (2.1 to 6.3)	3.2 (1.9 to 4.7)
5ASA therapy	39 (31)	10 (12)	30 (65)
AZA therapy	56 (44)	38 (46)	18 (39)
MTX therapy	29 (23)	25 (30)	4 (8.7)
Infliximab therapy	42 (33)	33 (40)	9 (20)
Adalimumab	3 (2.4)	2 (2.4)	1 (2.1)

5ASA monotherapy	22 (17)	2 (2.4)	20 (43)

AZA monotherapy w/o 5ASA	26 (20)	24 (29)	3 (6.5)
AZA monotherapy w 5ASA	12 (9.4)	4 (4.8)	9 (20)
AZA monotherapy w or w/o 5ASA	38 (30)	27 (33)	11 (24)

MTX monotherapy w/o 5ASA	8 (6.7)	8 (10)	2 (4.3)
MTX monotherapy w 5ASA	2 (1.6)	2 (2.4)	0 (0.0)
MTX monotherapy w or wo 5ASA	10 (79)	9 (11)	2 (4.3)

Infliximab monotherapy	7 (5.5)	7 (8.5)	0 (0.0)
Infliximab & AZA	18 (14)	11 (13)	7 (15)
Infliximab & AZA & 5ASA	4 (3.1)	2 (2.4)	3 (6.5)
Infliximab & MTX	17 (13)	15 (18)	2 (4.3)
Infliximab & MTX & 5ASA	2 (1.6)	1 (1.2)	1 (2.1)

Adalimumab monotherapy	1 (0.7)	0 (0.0)	1 (2.1)
Adalimumab & MTX	2 (1.6)	2 (2.4)	0 (0.0)

^*∗*^1 subject has IBD-U. No patient on biologics and ASA alone. No patients on Adalimumab and AZA or Adalimumab and ASA alone.

**Table 2:** Adherence rates.

Therapy	*N* observations per year over 2 years	Median adherence rate	IQR (25–75)
*Immunomodulators*			
Total group	160	88%	58 to 100
Crohn's	120	88%	58 to 100
UC	40	87%	58 to 100
*5-ASA*			
Total group	72	82%	49 to 100
Crohn's	18	92%	46 to 100
UC	54	81%	49 to 100
*Anti-TNFα*			
Total group	80	100%	83 to 100
Crohn's	63	100%	82 to 100
UC	17	100%	86 to 100
*Blood work*			
Total group	250	67%	42 to 100
Crohn's	159	67%	42 to 92
UC	91	67%	33 to 100

**Table:** (a) Univaried description of independent categorical variables and adherence

Categorical variables	Immunomodulators		5-ASA		Biologics		Blood work adherence	
	Number of observations per yr over 2 years	Number with >80% adherence rate (%)	Number of observations per yr over 2 years	Number with >80% adherence rate (%)	Number of observations per yr over 2 years	Number with >80% adherence rate (%)	Number of observations per yr over 2 years	Number with >80% adherence rate (%)
Total	160	91 (57)	72	37 (51)	80	65 (81)	250	102 (41)
Sex								
Female	69	38 (55)	37	20 (54)	22	20 (91)	109	42 (39)
Male	91	53 (58)	35	17 (49)	58	45 (76)	141	60 (43)
Disease type								
Crohn's	120	68 (57)	18	10 (56)	63	51 (81)	159	64 (40)
Ulcerative colitis	40	23 (58)	54	27 (50)	17	14 (82)	91	38 (41)
Disease activity severity								
Mild	52	29 (56)	31	11 (35)	14	11 (79)	92	36 (39)
Moderate	81	46 (57)	31	20 (65)	44	35 (80)	112	49 (44)
Severe	27	16 (59)	10	6 (60)	21	18 (86)	44	15 (34)
Exacerbation								
No	126	74 (59)	52	28 (54)	64	52 (81)	202	76 (38)
Yes	34	17 (50)	20	9 (45)	16	13 (81)	48	26 (54)
Address								
Rural	57	31 (54)	30	10 (33)	24	21 (88)	88	29 (33)
Urban	103	60 (58)	42	27 (64)	56	44 (79)	162	73 (45)
Clinic visits per year								
0 (0-1 visit)	41	23 (56)	24	12 (50)	20	17 (85)	81	24 (30)
1 (2-3 visits)	41	21 (51)	18	9 (50)	17	16 (94)	66	27 (41)
2 (>3 visits)	78	47 (60)	30	16 (53)	43	32 (74)	103	51 (50)

**Table:** (b) Univaried description of adherence and independent continuous variables. Adherence is defined as taking more than 80% of medication

Continuous variables	Age (yrs) of adherent patients	Age (yrs) of nonadherent patients	Number of clinic visits per year of adherent patients	Number of clinic visits per year of nonadherent patients	Duration of disease (yrs) of those adherence	Duration of disease (yrs) of those nonadherent
*Immunomodulators*						
*n* observations	91	69	91	69	91	69
Median	11.9	11.5	3	2	2.3	3.64
IQR	9.06 to 13.80	8.80 to 13.32	1.00 to 4.00	1.00 to 4.00	1.90 to 4.35	1.30 to 7.25
Mean	10.95	10.59	2.88	2.79	2.88	2.8
std. Dev.	3.79	4.07	2.11	1.91	2.11	1.91
*5-ASA*						
*n* observations	37	35	37	35	37	35
Median	13.18	10.99	2	2	2.4	2.92
IQR	7.47, 14.45	6.88, 13.18	1.00 to 3.00	1.00 to 4.00	2.00 to 3.25	1.88 to 4.43
Mean	11.12	10.46	2.7	2.69	3.26	3.63
std. Dev.	4.87	3.74	2.25	2.21	2.25	2.6
*Biologics*						
*n* observations	65	15	65	15	65	15
Median	11.20	11.19	2.00	3.00	3.76	5.52
IQR	6.75 to 13.37	9.45 to 13.05	1.00 to 4.00	2.00 to 4.00	2.05 to 6.80	2.30 to 6.04
Mean	10.29	11.02	2.9	2.8	4.77	4.62
std. Dev.	4.14	2.48	2.06	1.47	3.26	1.98
*Blood work*						
*n* observations	102	148	102	148	102	148
Median	12.27	10.7	2.5	2	2.73	3.7
IQR	8.36 to 13.73	7.10 to 13.05	2.00 to 4.00	1.00 to 3.00	1.90 to 4.51	2.2 to 6.80
Mean	11.12	9.84	2.9	2.21	3.45	4.71
std. Dev.	3.9	4.01	1.89	1.82	2.33	3.03

**Table:** (a) Univariate analysis of factors that were associated with medications and blood work adherence. (1 = reference)

Variables	Univaried											
	Immunomodulators			5-ASA			Biologics			Blood work		
	odd	95% CI	*p* value	odd	95% CI	*p* value	odd	95% CI	*p* value	odd	95% CI	*p* value
Age												
Year	1.01	0.91, 1.11	0.83	1.04	0.91, 1.18	0.56	0.95	0.85, 1.06	0.37	1.09	1.00, 1,17	0.04
Sex												
Female	1			1			1			1		
Male	1.03	0.42, 2.51	0.95	0.8	0.29, 2.29	0.69	0.4	0.08, 1.65	0.19	1.1	0.64, 2.04	0.66
Disease type												
Crohn's	1			1			1			1		
Ulcerative colitis	1.02	0.43, 2.41	0.96	0.8	0.23, 2.55	0.67	1.1	0.30, 4.07	0.89	1.1	0.58, 1.95	0.84
Duration of disease												
Each year diagnosed	0.83	0.71, 0.97	0.02	0.9	0.76, 1.14	0.52	1	0.88, 1.17	0.81	0.8	0.73, 0.96	0.01
Disease severity												
Mild	1			1			1			1		
Moderate	1.07	0.44, 2.62	0.88	3.2	1.04, 9.88	0.04	1.1	0.28, 4.03	0.93	1.2	0.64, 2.34	0.54
Severe	1.12	0.39, 3.26	0.83	2.6	0.49, 13.6	0.26	1.6	0.23, 11.5	0.62	0.8	0.32, 1.99	0.63
Exacerbation												
No	1			1			1			1		
Yes	0.94	0.46, 1.94	0.88	0.7	0.22, 2.21	0.53	1.1	0.24, 4.63	0.94	2	1.08, 3.59	0.03
Address												
Rural	1			1			1					
Urban	1.09	0.49, 2.42	0.84	3.5	1.18, 10.56	0.02	0.5	0.13, 1.91	0.32	1.6	0.87, 3.40	0.125
Clinic visits per year												
0 (0-1 visit)	1			1			1			1		
1 (2-3 visits)	0.84	0.41, 1.56	0.58	1.2	0.32, 4.26	0.82	2.9	0.27, 3.16	0.38	1.13	0.58, 2.24	0.71
2 (>3 visits)	0.95	0.55, 1.63	0.39	1.1	0.30, 4.33	0.85	0.5	0.12, 2.06	0.34	1.30	0.70, 2.45	0.05

**Table:** (b) Multivariate analysis of factors that were significantly associated with medications and blood work adherence (1 = reference).

Multivariable analysis			
Variables	Odd	95% CI	*p* value
	Immunomodulators		

Disease duration			
Year	**0.82**	**0.70**,** 0.97**	**0.02**
Sex			
Female	1		
Male	**1.02**	**0.42, 2.51**	**0.06**

	5-ASA		

Disease severity			
Mild	1		
Mod	**3.2**	**1.09**,** 9.53**	**0.03**
Severe	3.0	0.46, 23.8	0.24
Address			
Rural	1		
Urban	**3.6**	**1.20**,** 11.0**	**0.02**

	Blood work		

Disease Duration			
Year	**0.86**	**0.74**,** 0.99**	**0.04**
Exacerbation			
	1		
	**1.7**	**0.92**,** 3.29**	**0.09**

**Table 5:** Adherence to medication predicts 6-month steroid-free remission rates (using Wilcoxon rank-sum (Mann–Whitney) test with steroid-free remission as dependent variable and adherence rate as independent variables).

	Steroid-free remission group		Steroid use relapse group		*Z*	*p* value
	Median adherence rate% (IQR)	*n* of observations	Median adherence rate% (IQR)	*n* of observations		
Immunomodulators	90 (62 to 90)	150	57 (42 to 85)	10	−2.56	0.01
5ASA	82 (49 to 100)	64	48 (41 to 79)	8	−1.34	0.18
Biologics	100 (83 to 100)	79	71 (70 to 71)	1	−1.6	0.11
Combined all 3 drugs	86 (67 to 100)	198	53 (42 to 85)	15	−2.67	0.008
Blood work	67 (42 to 96)	232	67 (50 to 100)	18	0.72	0.47

## Abbreviations

IBD: Inflammatory bowel disease
UC: Ulcerative colitis
IBD-U: Inflammatory bowel disease-unclassified
GEE: Generalized estimation equations
MTX: Methotrexate
AZA: Azathioprine
PCDAI: Pediatric Crohn's Disease Activity Index
PUCAI: Pediatric Ulcerative Colitis Activity Index
EPIC: Edmonton Pediatric IBD Clinic.

## References

[1] Gray W. N., Denson L. A., Baldassano R. N., Hommel K. A. (2012). Treatment adherence in adolescents with inflammatory bowel disease: The collective impact of barriers to adherence and anxiety/depressive symptoms. *Journal of Pediatric Psychology*.

[2] Fedorak R. N., Wong K., Bridges R. (2010). Canadian digestive health foundation public impact series. Inflammatory bowel disease in Canada: incidence, prevalence, and direct and indirect economic impact. *Canadian Journal of Gastroenterology & Hepatology*.

[3] Crohns and Colitis Foundation Annual Report. Crohns and Colitis Foundation of Canada. http://www.ccfa.ca.

[4] Hommel K. A., Denson L. A., Baldassano R. N. (2013). Oral medication adherence and disease severity in pediatric inflammatory bowel disease. *Inflammatory Bowel Diseases*.

[5] Greenley R. N., Kunz J. H., Walter J., Hommel K. A. (2013). Practical strategies for enhancing adherence to treatment regimen in inflammatory bowel disease. *Inflammatory Bowel Diseases*.

[6] Hall R. L., Willgoss T., Humphrey L. J., Kongso J. H. (2014). The effect of medical device dose-memory functions on patients' adherence to treatment, confidence, and disease self-management. *Patient Preference and Adherence*.

[7] Ma C., Evaschesen W., Gracias G. (2015). Inflammatory bowel disease patients are frequently non adherent to scheduled induction and maintenance therapy: A Canadian cohort study. *Canadian Journal of Gastroenterology and Hepatology*.

[8] Feagins L. A., Iqbal R., Spechler S. J. (2014). Case-control of factors that trigger inflammatory bowel disease flares. *World Journal of Gastroenterology*.

[9] Murphy G. K., McAlister F. A., Weir D. L., Tjosvold L., Eurich D. T. (2014). Cardiovascular medication utilization and adherence among adults living in rural and urban areas: a systematic review and meta-analysis. *BMC Public Health*.

[10] Kaspar K. (2013). A weighty matter: Heaviness influences the evaluation of disease severity, drug effectiveness, and side effects. *PLoS ONE*.

[11] Morgan C., McBeth J., Cordingley L. (2015). The influence of behavioural and psychological factors on medication adherence over time in rheumatoid arthritis patients: A study in the biologics era. *Rheumatology*.

[12] Sencan N. M., Wertheimer A., Levine C. B. (2011). What determines the duration of patient medication compliance in patients with chronic disease: Are we looking in the wrong place?. *Southern Med Review*.

[13] Cushing A., Metcalfe R. (2007). Optimizing medicines management: From compliance to concordance. *Therapeutics and Clinical Risk Management*.

[14] Horne R., Weinman J. (1999). Patients' beliefs about prescribed medicines and their role in adherence to treatment in chronic physical illness. *Journal of Psychosomatic Research*.

[15] Sylwestrzak G., Liu J., Stephenson J. J., Ruggieri A. P., DeVries A. (2014). Considering patient preferences when selecting anti-tumor necrosis factor therapeutic options. *American Health and Drug Benefits*.

